# Factors affecting cognitive function according to gender in community-dwelling elderly individuals

**DOI:** 10.4178/epih.e2017054

**Published:** 2017-11-15

**Authors:** Miwon Kim, Jeong-Mo Park

**Affiliations:** 1 Department of Nursing Science, Sangmyung University, Cheonan, Korea; 2 Department of Nursing, Kyungin Women’s University, Incheon, Korea

**Keywords:** Cognitive function, Elderly, Gender, Korea

## Abstract

**OBJECTIVES:**

This study aimed to identify the factors affecting the cognitive function of elderly people in a community by gender.

**METHODS:**

We obtained 4,878 secondary data of people aged ≥65 years in 2016 at a dementia prevention center in Gyeyang-gu, Incheon. Data were obtained through Mini-Mental Status Examination optimized for screening dementia and a questionnaire. The data were statistically analyzed using analysis of variance, analysis of covariance, and hierarchical regression.

**RESULTS:**

There were significant differences in cognitive function according to gender, and the differences were significant even when age was controlled, but gender differences disappeared when education was controlled. Age, education, social activities, number of comorbid diseases, and alcohol drinking affected cognitive function through interaction with gender, but interaction with gender disappeared when education was controlled. Regression analysis showed that depression, cohabitant, social activities etc., had a significant impact on both men and women under controlled education and age. In men, the effect of social activities was greater than that of women, and hyperlipidemia had the effect only in women.

**CONCLUSIONS:**

The differences in gender-related cognitive functions were due to differences in gender education period. The period of education is considered to have a great influence on cognitive function in relation to the economic level, occupation, and social activity.

## INTRODUCTION

In 2000, the population aged 65 years and older in South Korea (hereafter Korea) exceeded 7% of the total population and entered the aging society. As of 2017, the elderly population already exceeded 13.8% [1] and the elderly population is still rapidly growing. The rapid aging of the population has social and economic cost implications, resulting in personal and social burdens. Aging is also a direct cause of many degenerative diseases, including dementia, the most serious neurodegenerative disease, which our society has to face due to aging [2]. According to the 3rd Dementia management integration plan, the number of patients with dementia is expected to exceed 1 million in 2024 and 2 million in 2014 [3]. Also, the annual rate of progression to mild cognitive impairment (MCI) in normal subjects has been estimated at between 1-4% annually, and subjects with MCI have an annual risk of 12% of developing dementia [4]. The decline in cognitive function due to aging progresses slowly, making it difficult to pathologically determine the exact timing of onset of dementia. However, once it has progressed to dementia, it causes deterioration in the quality of life of patients and their families and huge medical expenses, leading to high economic burden at the individual and national levels. Therefore, countermeasures against this are urgently needed [2]. Since no effective drug and treatment for cognitive impairment and dementia is currently available, early detection of modifiable risk factors for cognitive impairment and prevention of cognitive impairment and delaying the onset of dementia through related early intervention is an important task of the national dementia management project [2]. In line with this, the Dementia screening project for the early detection of dementia has intensively been implemented at public health centers nationwide since 2010. As a result, the number of patients with mild cognitive impairment undergoing treatment increased from 24,602 in 2010 to 105,598 in 2014 with an annual increase rate of 43.9% [5].

Factors affecting cognitive impairment that have been identified so far include age, educational period, gender [6-10], health life factors such as drinking and smoking [7], depression [11], social factors such as social activity and occupation, history of disease, and body mass index (BMI) [12]. However, among these factors, age, education, and depression are consistently reported as risk factors for cognitive impairment, but these factors are not all consistent among studies. When cognitive decline is regarded as a continuous process from normal cognitive function to mild cognitive impairment and dementia, the identification and management of influential factors such as cognitive decline-related demographic characteristics, comorbid diseases and health habits may contribute to the delay or prevention of dementia [6]. However, previous studies on prediction models for cognitive function have been conducted in patients with cognitive impairment or dementia; thus, there is a limitation to generalizing the results of those studies [6]. In order to determine factors for preventing cognitive decline, it is also necessary to conduct studies involving the entire elderly population living in the community, including the elderly with normal cognitive function and cognitive decline.

Cognitive decline is expected in all elderly people rather than a specific group, and those with cognitive decline have a wide range of characteristics in addition to demographic characteristics including gender. Therefore, it is difficult to see that cognitive decline factors identified in the whole elderly population may have the same effects in the sub-elderly group. In order to prevent cognitive decline more effectively, it is necessary to manage factors which were found to be significantly associated with cognitive decline depending on the target population. The basic distinction in establishing intervention plans suited for target participants is gender, and it is thus necessary to identify factors affecting cognitive decline according to gender and to establish intervention plans based on such a gender difference. Many previous studies investigating factors for the cognitive decline have reported that a difference prevalence of cognitive decline in men and women was [6-9,10,13]. Kim et al. [14] compared cognitive function using the Korean version of Mini-Mental Status Examination optimized for screening dementia (MMSE-DS), the Korean version of the Consortium to Establish a Registry for Alzheimer’s Disease Assessment Packet(CERAD-K) and Korean-MMSE (K= MMSE) and reported that gender among education period, age and gender was found to have the greatest effect on cognitive function as measured by all the tools. However, studies identifying differences in influential factors according to gender are scarce and the results are inconsistent. Women are reported to have more cognitive decline than men [6,7].

Therefore, the present study aimed to investigate factors affecting cognitive function in the community-dwelling elderly, to identify factors affecting cognitive decline according to gender.

## MATERIALS AND METHODS

### Study design and participants

This is a cross-sectional study designed to investigate factors affecting cognitive function in the elderly using data obtained from elderly individuals aged 65 years or older who underwent early dementia screening at a dementia prevention center located in Gyeyang-gu, Incheon, Korea in 2016. The participants underwent dementia screening by examiners who visited homes, community centers and senior citizen centers.

### Instruments

#### Cognitive function-related variables

Age, gender, educational period, economic status, social life (past occupation, number of social activities, religion, cohabitant), history of disease (diabetes, hypertension, stroke, hyperlipidemia, number of comorbid diseases), and health habits (drinking, smoking, exercise) were examined.

#### Cognitive screening tool

Cognitive function state was examined using the MMSE-DS [14]. The reliability of this tool, as reported by Kim et al. [14] was Cronbach’s α= 0.826, and its reliability in the present study was Cronbach’s α= 0.839.

#### Depression scale

The degree of depression was assessed using the Short Form Geriatric Depression Scale (S-GDS)-Korean version of the 15-item S-GDS originally developed by Sheikh & Yesavage [15]. The Korean version was translated and standardized by Ki [16]. The scores range from 0 to 15 points on a 2-point scale (1 point for ‘yes’ and 0 point for ‘no’), and a higher score indicates a higher degree of depression. The degree of depression is classified as normal state for a score of less than 4 points, mild depression for a score of 5-8 points and severe depression for a score of more than 9 points. Its reliability in a study by Ki [16] was Cronbach’s α= 0.884.

### Data collection

For secondary data analysis, the present study obtained the consent from the head of the institution for the scope and contents of the data and was approved by the official institutional review board (IRB) designated by the Ministry of Health and Welfare Affairs (IRB no. PO1-201703-21-019). Data were collected through dementia screening as part of an early dementia screening, publicity and education project at a dementia prevention center from December 2015 to the end of December 2016. Dementia screening was performed by 4 nurses who completed a specialized dementia education and were systemically educated about the screening method. After all the participants were briefed about the purpose and contents of the screening and the present study and provided their consent for the use of the screening results, the screening and data collection were performed in a quiet place on a 1:1 basis.

### Data analysis

The data were obtained from 4,878 participants with complete entries among those who received dementia screening, and were analyzed using SPSS version 18.0 (SPSS Inc., Chicago, IL, USA). Differences in gender distribution of all the variables were analyzed using real numbers, percentages and chi-square test. Cognitive function by gender for each variable was analyzed using t-test and analysis of variance (ANOVA), the interaction of each variable with gender was analyzed using two way ANOVA, and the interactions of each variable with gender when age and education was controlled were analyzed using analysis of covariance. The factors affecting cognitive impairment were analyzed using hierarchical regression. The significance level for the statistical test was set to 0.05.

## RESULTS

### Distribution of cognitive function-related variables according to gender

We identified differences in the distribution of age, depression, education period, economic status, cohabitant, social life, history of disease, and lifestyle habits among the participants according to gender (Table 1).

### Gender comparison of cognitive function score according to each variable

The cognitive function scores by gender were 25.53±3.72 points in men and 23.76±4.47 in the women, showing a significant difference (F= 54.31; p< 0.001).

There were significant differences in cognitive function scores in the whole group, men participants and women participants according to age, education, depression, past occupation, economic status, cohabitant, drinking and exercise. There was no significant difference in cognitive function score according to the presence or absence of diabetes in the whole group, men and women participants (Table 2).

However, the cognitive function scores differed between the whole group, men and women participants according to number of social activities, religion, hypertension, hyperlipidemia, stroke, number of comorbid diseases and smoking (Table 2).

### Gender interaction with cognitive function-related factors

Two-way ANOVA was performed to determine the interaction of each variable affecting cognitive function with gender using gender and each variable as independent variables. As a result, the variables that interacted with gender in affecting cognitive function scores included age (F = 1.86; P < 0.01), education period (F= 3.56; p< 0.05), social activities (F= 1.94; p< 0.05), cohabitant (F = 4.15.15; p < 0.05), number of comorbid diseases (F = 2.74; p< 0.05) and drinking (F= 4.62, p< 0.01). Hypertension, stroke and smoking did not show gender interactions and the main effects were not significant, showing that hypertension, stroke and smoking did not affect cognitive function. Depression, past occupation, religion, economic status, hyperlipidemia and exercise showed significant main effects without interactions with gender (Table 3).

In assess for possible interactive variables after controlling for gender and other variables, two-way ANOVA between gender and each variable was performed by using age and education period as co-variates. When gender interactions with each variable and main effects when only age was controlled were identified, the results showed that hyperlipidemia, the number of comorbid diseases and drinking had significant interactions with gender (F= 4.28, p< 0.05; F= 3.57; p<0.05, F= 4.08; p<0.05), and all interactions with gender disappeared when only education was controlled (Table3).

### Factors affecting cognitive function according to gender

The aptness of the regression equation was found to be acceptable for men with a tolerance of 0.730-0.970, variance inflation factor (VIF) of 1.031-1.369 and Durbin-Watson statistic of 1.898 and for women with a tolerance of 0.732-0.973, VIF of 1.028-1.366 and Durbin-Watson statistic of 1.835.

To identify factors affecting cognitive function when controlling for education and age, education and age were first input and then the remaining variables were input to perform hierarchical regression analysis by gender (Table 4).

For the male participants, the effect of education (β= 0.30) and age (β= -0.17) on cognitive function scores was R^2^ = 0.14. The effects of depression (β= -0.16), living with non-spouse (β= -0.11), social activities (β = 0.10), economic status (β = -0.09), exercise (β= 0.08), presence or absence of stroke (β= 0.06) and presence or absence of religion (β= 0.06) on cognitive function score were low but significant.

For the female participants, the effect of education period (β=0.36) and age (β= -0.24) on cognitive function score was R^2^ = 0.25. Depression (β= -0.16), living with non-spouse (β= -0.08), hyperlipidemia (β= 0.05), economic status (β= -0.06), social activities (β= 0.04), exercise (β= 0.04), the presence or absence of religion (β= -0.03) and presence or absence of stroke (β= -0.03) had significant effects on cognitive function score.

## DISCUSSION

The proportion of elderly participants aged 85 years or older was higher among women, and the proportion of those with education period of less than 3 years was higher among women, which were similar to the distribution of age and education period in the studies by Park et al. [10], Kim et al. [14], and Park et al. [17] of community-dwelling elderly people. Furthermore, the results of the present study found that the proportion of those who engaged in regular exercise was higher among men, which has been reported as a cognitive function protective factor in previous studies [18,19], and smoking and drinking, which have been reported as cognitive function risk factors were more frequent in the men. From these results, it can be predicted that the difference in gender distribution of cognitive function protective factors or risk factors, or the relationship between those factors and gender might affect the gender differences in cognitive function.

Looking at cognitive function scores according to cognitive variables, possible risk factors for both genders included short education period, high depression, ‘no past occupation’ ‘living with nonspouse family members,’ ‘economic status’ and ‘no exercise’. Depression has been considered as a predictor of cognitive function in many previous studies [18,20,21]; it is therefore an important factor for maintaining cognitive function, and it is thought that continuous attention should be paid to depression to prevent decline in cognitive function. Living with family in terms of cohabitation type is accepted as a cognitive protective factor. Fratiglioni et al. [22] and Park et al. [17] reported that elderly individuals living alone had a relatively low cognitive function compared to those living with family, and explained that such results were due to the fact that elderly people living alone had a lack of emotional and cognitive stimulation and sense due to isolated life with little family and social ties. However, the present study found that elderly individuals living with their spouse had the highest cognitive function scores, followed by elderly people living alone and elderly people living with non-spouse family. This suggests that the cohabitation type of Korean elderly people is also changing to be couple-oriented, and living with children other than spouse is considered not to be protective of cognitive function compared to living alone. Continuous observational studies regarding cohabitation-related factors in Korean elderly people are needed.

Regarding drinking among lifestyle factors, ‘never-drinkers’ and ‘those who stopped drinking’ among the man participants had a lower cognitive function than the current drinkers, whereas the ‘current drinkers’ in the women group were found to have the lowest cognitive function score (22.33±5.25 points), suggesting that the effects of drinking on cognitive function may differ according to gender. The studies by Park & Song [8], Shin et al. [23], and Kim & Shim [24] have reported that drinking was associated with cognitive function; however, Topiwala et al. [25] reported through a systematic literature review that drinking was not associated with dementia. The results of the present study also showed that the effects of drinking on cognitive function were not clear. In this regard, it is necessary to investigate the degree and duration of drinking and to examine their relationship with other confounding variables. Men and women participants who exercised were found to have high cognitive function in the present study, which was consistent with the results of previous studies investigating the effects of physical activity on cognitive impairment [17,21]. Therefore, the present study confirmed that exercise was protective of cognitive function.

Vascular risk factors such as hypertension and diabetes are estimated to be risk factors for the progression of cognitive decline and transition from mild cognitive impairment to dementia [6,21, 26]. But the results of the present study showed that there was no difference in cognitive function according to diabetes and stroke, which were inconsistent with the results of previous studies [8,17, 21]. Further studies are needed to investigate the degree of hypertension and duration of disease.

The present study showed that only among woman participants, there were differences in cognitive function according to hyperlipidemia and there were a higher cognitive function in the woman participants with hyperlipidemia than those without hyperlipidemia, suggesting that hyperlipidemia may have a positive effect on cognitive function in women. In a study by Park et al. [17], hyperlipidemia was reported as a protective factor for mild cognitive impairment because it showed a negative relative risk and odds ratio, which were similar to the results of a study by Vidoni et al. [27] reporting that a low cholesterol or a low BMI acts as a risk factor for cognitive impairment.

There was a significant difference in cognitive function according to stroke only in man participants (F= 5.23; p< 0.05), showing that stroke was a risk factor for cognitive function among man participants, and this finding was similar to the results of a study by Park et al. [17] revealing that stroke had a greater relative risk in men than women.

The cognitive function according to smoking status was not different and the lowest cognitive function score was observed in never-smokers. These findings are similar to the results of studies by Shin et al.[23], and other researchers [10,23]. However, Kim [7] and Rakesh et al. [21] have reported that smoking was a risk factor for cognitive function. More detailed studies on the effects of smoking on cognitive function are thus needed.

Whether the gender difference in cognitive function as found in the present study was simply due to gender difference or gender difference in cognitive function-related factors or due to the results of interaction between those factors and gender is discussed as follows. Cognitive function scores did not differ according to gender when the education period was the same, and differences in cognitive function were found to be due to education period rather than age in the present study. This finding was consistent with the results of a study by Kim [7] and Park et al. [10] indicating that education was the most influential variable on cognitive function. Lin et al. [9] described that cognitive impairment and dementia were more likely to affect women than men, but this could be seen as a difference in age distribution. However, the results of the present study can be interpreted based on the explanation that cognitive impairment might progress faster as education period is shorter and the resulting cognitive simulation is lacking [28]. This is in line with the results of a study by Petersen et al. [29] stating that men might be relatively slower to progress from mild cognitive impairment to dementia because they have a relatively longer education period than women.

In addition, there were gender interactions with age, education period, social activities, cohabitant, drinking and the number of comorbid diseases among cognitive function-related variables in affecting cognitive function. However, when age was controlled, social activities and cohabitant showed no gender interaction, and only significant main effects were observed, suggesting that the difference in gender and age might affect cognitive function in relation to social activities and cohabitant. Meanwhile, drinking and the number of comorbid diseases showed significant interactions with gender even when age was controlled. However, when education was controlled, these variables did not show interactions with gender and the main effects of each variable were significant. The significant interactions of these variables with gender are interpreted to be due to gender differences in education period.

Finally, as a result of regression analysis it was found that depression in addition to education and age had a great effect on cognitive function in both gender participants (Table 4). The variable that differed between man and woman participants was found to be hyperlipidemia and was found to be a significant variable only in the woman participants. Therefore, further studies are needed to determine whether hyperlipidemia itself has a protective function for cognitive function, or whether it is related to education period and economic status in addition to age.

In conclusion, the results of the present study showed that fender differences in cognitive function was due to differences in education period. And factors affecting cognitive function were same for men and women, but, hyperlipidemia was added as a factor affecting cognitive function in the female participants.

Further studies are needed to investigate factors affecting cognitive function, the interactions with education and the combined effects. It is thought that it is effective to provide elderly individuals with interventional programs for improving cognitive function by segmenting them by education period and age.

## Figures and Tables

**Table 1.** Distribution of cognitive-related variables by gender

Variables	Categories	Total	Men	Women	χ^2^	p-value
Gender		4,878 (100.0)	1,110 (22.8)	3,768 (77.2)		
Age	<74	1,868 (38.3)	475 (42.8)	1,393 (37.0)	723.30	<0.001
	75-84	2,351 (48.2)	519 (46.8)	1,832 (48.6)		
	≥85	659 (13.5)	116 (10.5)	543 (14.4)		
Education period (yr)	0-3	1,803 (37.0)	113 (10.2)	1,690 (44.9)	13.58	<0.001
	4-6	1,519 (31.1)	321 (28.9)	1,198 (31.8)		
	7-12	1,365 (28.0)	552 (49.7)	813 (21.6)		
	≥13	188 (3.9)	124 (11.2)	64 (1.7)		
Depression	Normal	3,664 (75.1)	830 (74.8)	2,834 (75.2)	1.66	0.44
	Mild depression	638 (13.1)	138 (12.4)	500 (13.3)		
	Severe depression	576 (11.8)	142 (12.8)	434 (11.5)		
Past occupation	Never	2,819 (57.8)	179 (16.1)	2,640 (70.1)	1,022.60	<0.001
	Yes	2059 (42.2)	931 (83.9)	1,128 (29.9)		
No. of social activities	0	1,026 (21.0)	335 (30.2)	691 (18.3)	92.69	<0.001
	1	2,972 (60.9)	640 (57.7)	2,332 (61.9)		
	≥2	880 (18.0)	135 (12.2)	745 (19.8)		
Religion	No	1,665 (33.0)	533 (46.2)	2,636 (30.0)	127.98	<0.001
	Yes	3,213 (67.0)	577 (53.8)	574 (70.0)		
Cohabitant	Alone	1,514 (31.0)	209 (18.8)	1,305 (34.6)	593.98	<0.001
	Spouse	1,917 (39.3)	781 (70.4)	1,136 (30.1)		
	Non-spouse	1,447 (29.7)	120 (10.8)	1,327 (35.2)		
ES	National Health Insurance	4,604 (94.4)	1,031 (92.9)	3,573 (94.8)	6.28	0.01
	Medical Aid	273 (5.6)	79 (7.1)	194 (5.1)		
Hypertension	No	2,030 (41.6)	575 (51.8)	1,455 (38.6)	61.06	<0.001
	Yes	2,845 (58.4)	535 (48.2)	2,310 (61.3)		
DM	No	3,664 (75.1)	839 (75.6)	2,825 (75.0)	0.17	1.00
	Yes	1,214 (24.9)	271 (24.4)	943 (25.0)		
Hyperlipidemia	No	4,084 (83.7)	1,008 (90.8)	3,076 (81.6)	52.98	<0.001
	Yes	794 (16.3)	102 (9.2)	692 (18.4)		
Stroke	No	4,641 (95.1)	1,052 (94.8)	3,589 (95.2)	0.42	0.52
	Yes	237 (4.9)	58 (5.2)	179 (4.7)		
No. of comorbid diseases	0	833 (17.1)	255 (23.0)	578 (15.3)	51.78	<0.001
	1-2	3,209 (65.8)	716 (64.5)	2,493 (66.2)		
	≥3	836 (17.1)	139 (12.5)	697 (18.5)		
Alcohol drinking	Never	3,618 (74.2)	239 (21.5)	3,379 (89.7)	2,105.34	<0.001
	Ceased drinking	552 (11.3)	420 (37.8)	132 (3.5)		
	Currently drinking	708 (14.5)	451 (40.6)	257 (6.8)		
Smoking	Never	3,995 (81.9)	329 (29.6)	3,666 (97.3)	2,659.31	<0.001
	Ceased smoking	705 (14.4)	641 (57.7)	64 (1.7)		
	currently smoking	178 (3.6)	140 (12.6)	38 (1.0)		
Exercise	No	2,264 (46.4)	418 (37.7)	1,846 (49.0)	44.28	<0.001
	Yes	2,614 (53.6)	692 (62.3)	1,922 (51.0)		

Values are presented as number (%). ES, economic status by health insurance type; DM, diabetes mellitus.

**Table 2.** Gender comparison of cognitive function score differences by variables

Variables	Categories	Total		Men		Women	
		Mean±SD	t or F	Mean±SD	t or F	Mean±SD	t or F
Gender	Men	25.53±3.72	54.31^***^				
	Women	23.76±4.47					
Age	<74	25.17±3.41	303.29^***^	26.32±3.20	25.161^***^	25.56±2.46	202.99^***^
	75-84	26.64±4.32		25.04±3.84		23.25±4.37	
	≥85	23.16±4.60		24.25±4.34		20.86±5.06	
Education period (yr)	0-3	21.66±4.49	458.35^***^	22.73±4.15	49.99^***^	21.58±4.50	342.63^***^
	4-6	24.71±3.94		24.53±4.28		24.76±3.84	
	7-12	26.47±2.89		26.40±2.97		26.52±2.84	
	≥13	26.92±2.66		25.53±3.72		27.13±2.82	
Depression	Normal	24.62±4.00	95.83^***^	26.00±3.18	29.00^***^	24.21±4.12	73.89^***^
	Mild	23.23±4.68		24.43±4.43		22.89±4.69	
	Severe	22.00±5.67		23.69±5.16		21.45±5.72	
Past occupation	Never	23.40±4.64	208.76^***^	24.53±4.69	15.71^***^	23.33±4.63	83.18^***^
	Yes	25.20±3.73		25.73±3.47		24.76±3.88	
No. of social activities	0	23.90±4.95	2.70^+^	24.84±4.23	5.78^***^	23.44±5.20	2.85
	1	24.26±4.24		25.86±3.33		23.82±4.36	
	≥2	24.13±4.07		25.71±3.92		23.84±4.04	
Religion	No	24.29±4.43	10.58^***^	25.53±4.09	0.03	24.02±4.13	8.56^**^
	Yes	23.61±4.44		25.54±3.68		23.13±4.69	
Cohabitant	Alone	23.99±4.14	206.27^***^	25.35±3.54	17.62^***^	23.78±4.19	130.93^***^
	Spouse	25.51±3.55		25.86±3.45		25.27±3.59	
	Non-spouse	22.55±4.98		23.74±5.00		22.44±4.97	
ES	National Health Insurance	24.25±4.27	38.17^***^	25.64±3.58	12.98^***^	23.85±4.37	33.23^***^
	Medical Aid	22.58±5.60		24.09±5.02		21.96±5.72	
Hypertension	No	24.33±4.38	5.07^*^	25.46±3.69	0.43	23.88±4.54	1.79
	Yes	24.04±4.36		25.61±3.76		23.68±4.14	
DM	No	24.17±4.38	0.12	25.50±3.79	0.33	23.78±4.47	0.29
	Yes	24.13±4.34		25.65±3.51		23.69±4.46	
Hyperlipidemia	No	23.98±4.45	41.59^***^	25.48±3.72	2.33	23.49±4.56	58.98^***^
	Yes	25.07±3.83		26.07±3.73		24.93±3.83	
Stroke	No	24.18±4.36	1.46	25.59±3.70	5.23^*^	23.76±4.45	0.16
	Yes	23.83±4.63		24.45±3.97		23.63±4.82	
No. of comorbid diseases	0	24.37±4.45	0.81	25.25±3.73	12.97^***^	23.98±4.68	33.23^***^
	1-2	24.11±4.36		26.70±3.61		23.62±4.45	
	≥3	24.18±4.33		25.09±4.35		24.00±4.30	
Alcohol drinking	Never	23.84±4.47	45.13^***^	25.15±4.23	6.84^**^	23.75±4.47	10.85^***^
	Ceased drinking	24.53±4.36		26.03±3.30		24.55±3.72	
	Currently drinking	25.49±3.53		25.22±3.79		22.33±5.25	
Smoking	Never	23.90±4.41	40.43^***^	25.37±3.75	0.43	23.77±4.44	0.66
	Ceased smoking	25.42±3.92		25.61±3.72		23.56±5.24	
	Currently smoking	25.02±4.21		25.57±3.68		22.97±5.35	
Exercise	No	23.29±4.78	172.78^***^	24.68±4.41	36.94^***^	22.98±4.80	113.62^***^
	Yes	24.91±3.83		26.05±3.13		24.51±3.98	

ES, economic status by health insurance type; DM, diabetes mellitus.

+ p<0.1,

* p<0.05,

** p<0.01,

*** p<0.001.

**Table 3.** Gender interaction in the effect of variables on cognitive function

Variables	Source of variation	Gender*Variable	CV: Age	CV: Education
		F	Gender*Variable	Gender*Variable
			F	F
Age	(CV) Age	-	687.45^***^	-
	(CV) Education	-	-	683.20^***^
	Age	10.14^***^	-	6.31^***^
	Gender	44.68^***^	130.18^***^	2.56
	Gender*Age)	1.86^**^	-	1.11^+^
Education period	(CV) Age	-	270.07^***^	-
	(CV) Education	-	-	1,099.20^***^
	Education	153.55^***^	104.97^***^	-
	Gender	403.00	2.98^+^	0.09
	Gender*Education	3.56^*^	1.82	-
Depression	(CV) Age	-	743.92 ^***^	-
	(CV) Education	-	-	1,038.37^***^
	Gender	60.68^***^	53.46^***^	3.28^+^
	Depression	10.93^***^	14.30^***^	8.03^***^
	Gender*Depression	1.03	0.91	1.17
Past occupation	(CV) Age	-	599.32^***^	-
	(CV) Education	-	-	1,017.58^***^
	Gender	32.58^***^	41.15^***^	5.81^*^
	Past occupation	47.94^***^	17.35^***^	21.06^***^
	Gender*Past occupation	0.39	1.16	0.17
No. of social activities	(CV) Age	-	774.23^***^	-
	(CV) Education	-	-	1,097.86^***^
	Gender	2.96	6.08^*^	1.23
	No. of social activities	2.99^***^	8.00^***^	4.82^***^
	Gender* No. of social activities	1.94^**^	1.17	1.38
Religion	(CV) Age	-	700.72^***^	-
	(CV) Education	-		1,063.98^***^
	Gender	40.60^***^	40.90^***^	0.15
	Religion	7.86^***^	9.89^***^	5.84^***^
	Gender*Religion	2.11	1.64	2.12^+^
Cohabitant	(CV) Age	-	453.65^***^	-
	(CV) Education	-	-	896.60^***^
	Gender	40.85^***^	44.52^***^	3.69^***^
	Cohabitant	66.10^***^	23.90^***^	32.10^***^
	Gender*Cohabitant	4.15^*^	0.04	0.86
ES	(CV) Age	-	677.74^***^	
	(CV) Education	-		1,108.41^***^
	Gender	43.74^***^	26.52^***^	0.30
	ES	33.86^***^	34.19^***^	39.40^***^
	Gender*ES	3.18	0.52	0.45
Hypertension	(CV) Age	-	697.34^***^	
	(CV) Education	-		1,095.40^***^
	Gender	140.78^***^	137.48^***^	0.05
	Hypertension	0.04	5.33^*^	0.51
	Gender*Hypertension	1.40	0.89	0.01
Hyperlipidemia	(CV) Age	-	657.99^***^	-
	(CV) Education	-	-	1,079.49^***^
	Gender	42.48^***^	32.99^***^	1.03
	Hyperlipidemia	17.75^***^	6.90^**^	12.20^***^
	Gender*Hyperlipidemia	3.08^+^	4.28^*^	3.06^+^
Stroke	(CV) Age	-	689.25^***^	-
	(CV) Education	-	-	1,098.46^***^
	Gender	15.76^***^	16.28^***^	0.90
	Stroke	3.69^+^	5.87^*^	3.81^+^
	Gender*Stroke	2.27	1.26	0.84
No. of comorbid diseases	(CV) Age	-	696.34^***^	-
	(CV) Education	-	-	1,097.85^***^
	Gender	105.45^***^	93.99^***^	0.78
	No. of comorbid diseases	0.17	2.07	0.56
	Gender*No. of comorbid diseases	2.74^*^	3.57^*^	2.12^+^
Alcohol drinking	(CV) Age	-	667.61^***^	-
	(CV) Education	-	-	1,093.93^***^
	Gender	87.52^***^	95.52^***^	1.01
	Alcohol drinking	16.39^***^	6.52^***^	14.83^***^
	Gender*Alcohol drinking	4.62^**^	4.08^*^	3.01
Smoking	(CV) Age	-	689.75^***^	-
	(CV) Education	-	-	1,100.74^***^
	Gender	38.97^***^	29.03^***^	1.04
	Smoking	0.27	1.60	0.67
	Gender*Smoking	0.89	0.01	1.52
Exercise	(CV) Age	-	616.97^***^	-
	(CV) Education	-	-	1,003.71^***^
	Gender	119.45^***^	107.50^***^	0.27
	Exercise	94.69^***^	61.56^***^	46.25^***^
	Gender*Exercise	0.31	0.11	0.01

CV, co-variable; ES, economic status by health insurance type.

+ p<0.1,

* p<0.05,

** p<0.01,

*** p<0.001.

**Table 4.** Factors influencing cognitive function according to gender

Gender	Variables	Model 1					Model 2				
		B	SE	β	t	p-value	B	SE	β	t	p-value
Men	(Constant)	28.93	1.26		23.04	<0.001	28.39	1.40		20.28	<0.001
	Education period	1.38	0.13	0.30	10.65	<0.001	1.20	0.13	0.26	9.44	<0.001
	Age	0.09	0.02	-0.17	-6.10	<0.001	-0.10	0.02	-0.19	-6.51	<0.001
	Depression						-0.17	0.03	-0.16	-5.43	<0.001
	Cohabitant: non- spouse						-1.32	0.33	-0.11	-3.99	<0.001
	No. of social activities						0.56	0.16	0.10	3.51	<0.001
	ES						-1.23	0.41	-0.09	-3.03	0.002
	Exercise						0.60	0.21	0.08	2.80	0.005
	Stroke						-1.05	0.48	-0.06	-2.22	0.03
	Religion						0.42	0.21	0.06	2.03	0.04
		F(p)= 89.35^***^					F=18.83^***^				
		R^2^=0.14, adjusted R^2^=0.14, ΔR^2^=0.14					R^2^=0.23, adjusted R^2^=0.21, ΔR^2^=0.09				
Women	(Constant)	31.39	0.79		39.58	<0.001	31.12	0.90		34.72	<0.001
	Education period	1.92	0.08	0.36	23.28	<0.001	1.72	0.08	0.32	20.96	<0.001
	Age	-0.14	0.01	-0.24	-15.43	<0.001	-0.14	0.01	-0.23	-13.38	<0.001
	Depression						-0.21	0.02	-0.16	-10.93	<0.001
	Cohabitant: non- spouse						-0.74	0.17	-0.08	-4.39	<0.001
	ES						-1.30	0.28	-0.06	-4.65	<0.001
	Hyperlipidemia						0.63	0.19	0.05	3.29	0.001
	No. of social activities						0.31	0.10	0.04	3.08	0.002
	Exercise						0.33	0.13	0.04	2.55	0.01
	Religion						-0.33	0.14	-0.03	-2.37	0.02
	Stroke						-0.63	0.30	-0.03	-2.09	0.04
		F(p)=635.99^***^					F=99.00^***^				
		R^2^=0.25, adjusted R^2^=0.25, ΔR^2^=0.25					R^2^=0.31, adjusted R^2^=0.31, ΔR^2^=0.06				

SE, standard error; ES, economic status by health insurance type.

*** p<0.001.
