# Supplementary material for: Factors affecting cognitive function according to gender in community-dwelling elderly individuals
Source: Epidemiol Health. 2017 Nov 15;39:e2017054. doi: 10.4178/epih.e2017054 (PMC5790979; doi:10.4178/epih.e2017054)
Supplement: Supplementary file 1 [file epih-39-e2017054-supplementary.pdf]

Volume: 39, Article ID: e2017054

https://doi.org/10.4178/epih.e2017054

# Factors affecting cognitive function according to fender in community-dwelling elderly individuals

Miwon Kim[1], Jeong-Mo Park[2]

[1]Department of Nursing Science, Sangmyung University, Cheonan, Korea; [2]Department of Nursing, Kyungin Women's University, Inchon, Korea

## Abstract

**OBJECTIVES:** This study aimed to identify the factors affecting the cognitive function of elderly people in a community by gender.
**METHODS**: We obtained 4,878 secondary data of people aged ≥65 years in 2016 at a dementia prevention center in Gyeyang-gu, Incheon. Data were obtained through Mini-Mental Status Examination optimized for screening dementia and a questionnaire. The data were statistically analyzed using analysis of variance, analysis of covariance, and hierarchical regression.
**RESULTS:** There were significant differences in cognitive function according to gender, and the differences were significant even when age was controlled, but gender differences disappeared when education was controlled. Age, education, social activities, number of diseases, and alcohol drinking consumption affected cognitive function through interaction with gender, but interaction with gender disappeared when education was controlled. Regression analysis showed that depression, cohabitant, social activities etc., had a significant impact on both men and women under controlled education and age. In men, the effect of social activities was greater than that of women, and hyperlipidemia had the effect only in women.
**CONCLUSIONS:** The differences in gender-related cognitive functions were due to differences in gender education period. The period of education is considered to have a great influence on cognitive function in relation to the economic period, occupation, and social activity.

Key Words: Cognitive function, Elderly, Gender

https://doi.org/10.4178/epih.e2017054

# 서론

우리나라는 2000년에 65세 인구가 7%를 넘어 고령화 국가로 진입한 이래로 2017년 현재 이미 노인인구가 13.8%를 넘어[1] 고령인구가 빠르게 증가하고 있다. 급속한 노령화는 사회, 경제적 비용의 증가로 개인적, 사회적 부담을 초래하게 된다. 인구의 고령화는 여러 가지 퇴행성질환의 발병에 직접적인 원인으로 작용하는데, 그 중에서도 인지기능 저하는 고령화로 인해 우리사회가 감당해야 할 가장 심각한 퇴행성뇌신경질환인 치매로 진행되는 중요한 문제이다[2]. 제 3차 치매관리종합계획수립연구에 의하면 65세 이상 노인인구 중 치매환자 수가 2024년에는 100만, 2041년에는 이백만을 넘어설 것으로 예상하고 있다[3]. 또한 정상 노인에 있어MCI 진행률은 연 1~4%로 추정되며 MCI 환자는 매년 12%가 치매 로 진전될 위험험이 있다[4]. 노화에 따른 인지 기능의 저하는 수년에 걸쳐 서서히 진행됨에 따라 치매발병시점을 병리학적으로 정확하게 확인하기 어렵다. 그러나 치매로 진행된 후에는 환자 본인과 가족의 삶의 질적 저하는 물론이고 막대한 의료비 지출로 인해 개인적 및 국가적 차원에서 심각한 수준의 경제적 부담으로 이어지기 때문에 이에 대한 대비책이 절실한 실정이다[2]. 현재로서는 인지 장애 및 치매로 진행된 후에는 이에 대한 효과적인 약물이나 치료법이 없으므로 수정 가능한 인지기능 저하 위험요인을 조기발견하고 이에 대한 조기개입을 통해 인지기능저하를 예방하고 치매발생을 지연시키는 것이 국가의 치매관리사업에서 중요한 과제이다[2]. 이에 따라 2010년부터 전국 보건소에서 치매조기발견을 위한 치매검진사업이 집중적으로 시작되었고 그 결과 경도인지장애 진료환자수가 2010년 24,602명에서 2014년 105,598명으로 연평균 43.9%가 증가하였다[5].

지금까지 밝혀진 인지장애 영향요인으로는 연령, 교육기간, 성별[6, 7, 8, 9, 10] 외에도 음주, 흡연 등 건강생활요인[7], 우울[11] 사회활동, 직업 등 사회적 요인, 병력, 체질량지수(BMI, Body Mass Index) 등을[12] 들 수 있다. 그러나 이중에서 일관적으로 인지저하를 초래하는 위험요인으로 보고되고 있는 것은 연령, 교육, 우울 등이며 이 요인들도 연구에 따라 모두 일치하고 있지는 않다. 인지기능저하를 정상인지기능 상태에서 경도인지장애를 거쳐 치매로 이르는 연속적인 과정으로 볼 때 인지저하 관련 인구학적 특성, 동반질환, 건강습관과 같은 영향요인을 확인하고 관리하면 치매발생의 지연 또는 예방에 기여할 수 있을 것이다[6]. 그러나 인지기능 예측모형에 관한 기존의 연구들은 주로 인지장애, 또는 치매환자를 대상으로 개발되었으므로 일반지역사회 인구에 적용하기에는 제한이 있다[6]. 따라서 인지기능 저하를 예방하기 위한 요인을 파악하기 위해서는 인지기능 정상부터 저하까지 포함된 지역사회 거주 전체 노인인구를 대상으로 한 연구도 필요하다.

Volume: 39, Article ID: e2017054

https://doi.org/10.4178/epih.e2017054

인지기능 저하는 특정 집단이 아닌 모든 노인에서 예상되는 것이며 대상자들은 성별을 포함한 인구학적 특성 외에도 매우 다양한 범위를 가지고 있으므로 전체 노인 집단을 대상으로 파악된 인지기능 저하 요인들이 하위 노인 집단에서 동일한 영향을 미친다고 보기 어렵다. 따라서 좀 더 효과적인 인지기능 저하 예방을 위해서는 인지기능 저하와 유의한 관계가 있다고 밝혀진 요인을 토대로 각 대상자에 가장 맞는 요인관리가 필요하다. 대상자에 맞는 중재 계획을 수립함에 있어 기본적인 구분은 성별임으로 성별에 따른 인지기능 저하 영향 요인을 확인하여 차이가 있다면 이를 반영하여 중재계획을 세우는 것이 필요하겠다. 그 동안 인지기능 저하 요인을 확인한 여러 선행연구에서 여성과 남성의 유병률의 차이가 있다는 보고가 다수 있으며[6, 7, 8, 9,10, 13] Kim 등[14]은 Korean version of Mini-Mental Status Examination optimized for screening dementia) (MMSE-DS), Korean Version of the Consortium to Establish a Registry for Alzheimer's Disease Assessment Packet(MMSE-KC), and Korean-MMSE(K-MMSE)로 인지기능을 비교한 결과 모든 도구에서 인지기능에 교육기간, 연령, 성별 중에서 성별이 가장 큰 영향을 미치고 있다고 보고하고 있다. 여성이 남성에 비해 인지기능저하가 더 많다고 보고되고 있으나[6,7]성별에 따른 명확한 영향요인의 차이를 밝힌 연구는 드물며 어떤 요인들이 성별의 인지기능 차이에 영향을 주는지에 대해서는 명확히 설명되지 못하고 있다.

따라서 본 연구는 지역사회 재가노인을 대상으로 인지기능 영향요인을 확인하고 인지기능의 성별 차이가 무엇에 기인하는지, 이들 요인이 성별에 따라 어떻게 영향을 미치는지를 확인하기 위해 시도되었다.

## 연구대상 및 방법

### 연구설계 및 대상

본 연구는 I시 G구 치매통합지원센터에서 치매조기검진을 받은 데이터를 이용하여 노인의 인지기능의 영향요인을 파악하기 위한 횡단적 조사연구이다. 본 연구의 대상자는 2016년 I시 G구에 거주하는 65세 이상 노인으로 치매통합지원센터를 방문한 노인 그리고 가정방문, 복지관, 경로당 등에 검진자가 방문하여 검진을 받은 노인들이다.

### 연구도구

*인지기능 관련 변수*

https://doi.org/10.4178/epih.e2017054

연령, 성별, 교육기간, 경제수준, 사회생활(과거직업, 사회활동 수, 종교, 동거형태), 질병력(당뇨, 고혈압, 뇌졸중, 고지혈증, 보유질환 수), 건강생활습관(음주, 흡연, 운동)에 대해 조사하였다.

*인지기능 선별검사 도구*

인지기능검사는 간이정신상태검사(MMSE-DS)[14]를 사용하여 인지기능상태를 검사하였다. Kim 등이 [14]가 보고한 이 도구의 신뢰는 Cronbach's α=. 826였으며, 본 연구에서의 신뢰도는 Cronbach's α=.839이었다.

*우울 검사 도구*

우울정도는 Sheikh와 Yesavage [15] 개발한 15개 단축형 우울측정척도(Short Form Geriatric Depression Scale, S-GDS)를 Ki [16]가 우리말로 번안하여 한국판 단축형 노인 우울척도로 표준화한 것을 사용하였다. 이 도구는 2점 척도로(예 1점, 아니오 0점) 점수의 범위는 0점- 15점까지며 점수가 높을수록 우울정도가 높은 것으로 의미한다. 우울수준은 4점 이하는 정상, 5점-8점은 경도우울, 9점 이상은 중증 우울로 분류하고 있다. Ki [16]의 연구에서 신뢰도는 Cronbach's α=.884였다.

**자료수집**

이차자료분석을 위해 자료사용범위와 내용에 대하여 기관장의 동의를 얻어 보건복지부 지정 공용기관생명윤리위원회의 승인을(IRB no. PO1-201703-21-019) 받았다. 자료수집은 2015년 12월부터 2016년 12월 말까지 I시 일개 구 치매통합지원센터에서 시행한 치매조기검진, 홍보 및 교육사업으로 시행된 선별검사를 통해 이루어졌다. 선별검진은 치매전문교육을 이수하고 검진방법을 체계적으로 교육받은 간호사 4명이 시행하였다. 검진과 자료수집은 대상자와 1:1 검진이 가능한 조용한 곳에서 검진과 조사의 목적과 내용을 설명하고 검진 및 조사결과 이용에 대한 동의를 받은 후 시행하였다.

**자료분석**

분석에 사용된 자료는 선별 검진한 자료 중 완전하게 기입된 4,878명의 자료이며, SPSS/WIN 18.0 프로그램을 이용하여 분석하였다. 제 변수의 성별 분포 차이는 실수, 백분율, Chi-square test를 사용하여 분석하였으며 각 변수에 따른 성별 인지기능은 t-test와 ANOVA를 사용하였고, 성별과 제 변수의 상호작용은 Two Way ANOVA를, 그리고 연령과 교육을 통제한 상태에서 제 변수와 성별과의 상호작용은 ANCOVA를 사용하였다. 인지장

https://doi.org/10.4178/epih.e2017054

애 영향요인은 위계적 회귀분석(hierarchical regression)을 실시하여 분석하였다. 본 연구에서 통계 결과 해석에 적용된 유의수준은 0.05이다.

## 연구결과

### 성별에 따른 인지기능 관련 변수 분포

연구대상의 연령, 성별, 우울, 교육기간, 경제수준, 동거형태, 사회생활(과거직업, 사회활동 수, 종교, 동거형태), 질병력(당뇨, 고혈압, 뇌졸중, 고지혈증, 보유질환 수), 건강생활습관(음주, 흡연, 운동)의 성별에 따른 분포의 차이를 확인한 결과는 <Table 1>과 같다.

### 각 변수에 따른 인지기능 점수의 성별 비교

성별 인지기능 점수는 남성은 25.53±3.72, 여성은 23.76±4.47으로 유의한 차이가 있었다(F=54.31, p<.001). 연령, 교육, 우울, 과거직업, 경제수준, 동거형태, 음주, 운동에 따라서는 전체집단, 남성, 여성 모두에서 인지기능 점수에 유의한 차이를 보였다. 당뇨유무는 전체, 남성, 여성 모두에서 유의한 차이를 보이지 않았다. 그러나 사회활동수, 종교, 고혈압, 뇌질환, 질병수, 흡연은 전체집단의 결과와 성별에서의 결과가 다르게 나타났다(Table 2).

### 인지기능 관련 요인과 성별 상호작용

본 연구에서는 인지기능의 성별차이가 단순히 성별에 따른 차이인지, 성별과 관련되는 다른 요인들의 영향 때문 인지를 확인하고자 하였다. 이를 위해 먼저 인지기능에 영향을 미치는 각 변수와 성별과 상호작용을 확인하고자 성별과 각 변수를 독립변수로 하여 이원분산분석을 시행하였다. 그 결과 인지기능 점수에 영향을 미침에 있어 성별과 상호작용이 있는 변수는 연령(F=1.86, p<0.01), 교육기간(F=3.56, p<0.05), 사회활동(F=1.94, p<0.01), 동거형태(F=4.15, p<0.05), 질병 수(F=2.74, p<0.05)로, 음주(F=4.62, p<0.01)로 나타났다. 고혈압, 뇌졸중, 흡연은 성별 상호작용이 나타나지 않을 뿐 아니라 주효과가 유의하지 않아 인지기능에 영향을 미치지 않는 것으로 나타났다. 우울, 과거직업, 종교, 경제수준, 고지혈증, 운동은 성별과의 상호작용 없이 유의한 주효과를 나타내었다(Table 3).

이어서 선행연구에서 인지기능에 큰 영향을 미치며 성별에 따른 차이가 있는 것으로 확인된 연령과 교육기간을 통제한 상태에서도 성별과 상호작용하는 변수들이 있는지 확인하기 위해 연령을 공변인으로, 그리고 교

https://doi.org/10.4178/epih.e2017054

육기간을 공변인으로 한 후에 성별과 각 변수간의 이원분산분석을 시행하였다. 연령을 통제한 상태에서 제 변수들과 성별의 상호작용과 주작용을 확인하였을 때에는 고지혈증, 질병수와 음주만이 성별과 유의한 상호작용을 보였으며($F=3.57$, $p<0.05$, $F=4.08$, $p<0.05$) 교육을 통제하였을 때에는 성별과의 상호작용이 모두 사라졌다.

## 성별 인지기능 영향요인

회귀식의 적합성을 확인한 결과 남성의 경우 공차 .730-.970, VIF 1.031 –1.369, Durbin-Watson 1.898, 여성의 경우 공차 .732-.973, VIF 1.028-1.366, Durbin-Watson로 1.835로 적합하였다.

교육과 나이를 통제한 상태에서 성별 인지기능에 영향을 미치는 요인을 확인하기 위해 교육과 나이를 먼저 투입하고 이어서 나머지 모든 변수를 투입하여 위계적 회귀분석을 각 성별로 시행하였다(Table 4). 남성의 경우 교육(β=.30), 연령(β=-.17)이 인지기능점수에 미치는 영향은 $R^2$=.14였고, 우울(β=-.16), 배우자외의 사람과 동거(β=-.11), 사회활동(β=.10), 경제수준(β=-.09), 운동(β=.08), 뇌졸중유무(β=.06), 종교유무(β=.06)는 영향정도는 낮으나 유의한 영향을 미치는 것으로 나타났다. 반면 여성의 경우 교육기간(β=.36), 연령(β=-.24)이 미치는 영향은 $R^2$=.25이었고, 우울(β=-.16), 배우자외의 사람과 동거(β=-.08), 고지혈증(β=.05), 경제수준(β=-.06), 사회활동(β=.04), 운동(β=.04), 종교유무(β=-.03)이 유의한 영향을 미치는 것으로 나타났다.

## 논의

본 연구대상자의 변수에 따른 분포를 볼 때 85세 이상 후기 고령인구비율은 여성이 많으며, 교육기간 3년이하 역시 여성이 많았는데 이 같은 결과는 Park등 [10], Kim 등[14], Park 등[17]이 지역사회 노인을 대상으로 한 연구에서의 연령 및 교육기간 분포와 유사하였다. 교육, 나이뿐 아니라 여러 선행연구[18, 19]에서 인지기능 보호요인으로 보고하고 있는 규칙적인 운동을 본 연구에서 남성이 더 많이 하고 있고, 인지기능 위험요인으로 보고되고 있는 음주와 흡연은 남성이 더 많이 하는 것을 볼 때 인지기능 보호요인, 또는 위험요인의 성별분포 차이, 또는 이들 요인들과 성별의 관계가 인지기능의 성별차이를 나타내는 결과에 영향을 미쳤을 것으로 예측할 수 있다.

인지기능 관련 변수들에 따른 인지기능의 차이는(Table 2) 남녀를 합한 전체집단에서의 결과와 성별 집단에서의 결과가 동일하지 않았는데 인지기능 관련 변수에 따른 인지기능점수를 살펴 볼 때 남녀모두에게 위험요

https://doi.org/10.4178/epih.e2017054

인으로 볼 수 있는 것은 짧은 교육기간, 높은우울, 과거직업 없음, 배우자 외의 가족과의 동거, 경제수준 건강보험대상자, 운동하지 않음이었다. 우울은 많은 선행연구[18, 20, 21]에서 인지기능 예측요인으로 보고 있다. 따라서 인지기능을 유지시킬 수 있는 중요한 요인으로 인지기능저하를 예방하기 위해서는 우울요인에 지속적으로 관심을 가져야 할 것으로 사료된다. 동거형태에서 가족과의 동거는 인지기능 보호요인으로 받아들여지고 있다. Fratiglioni 등[22], Park 등[17]은 독거노인이 가족동거노인에 비해 상대적으로 낮은 인지기능을 보이고 있음을 보고하면서 이러한 결과는 독거노인은 가족 및 사회와 유대관계가 적은 고립 된 생활로 인해 정서적, 인지적 자극과 감각이 결여되어 나타난 것으로 설명하고 있다. 그러나 본 연구에서는 전체, 남, 여 모두 부부와 동거하는 노인이 가장 인지기능점수가 높고 다음이 독거노인이며 다른 가족과 동거하는 노인이 가장 낮은 인지기능을 보여 독거가 인지기능에 부정적인 영향을 미치는 것으로 보고하고 있는 선행연구와 다른 결과를 나타냈다. 최근 Kim[7]의 연구에서도 독거가 인지기능에 유의하지 않은 것으로 나타났는데 이는 우리나라도 노년기 동거형태가 부부중심으로 변화하고 있고 배우자 외의 자녀등과의 동거가 혼자 거주하는 것 보다 인지기능보호요인으로 작용하고 있지 못하는 것으로 볼 수 있어 우리나라노인을 대상으로 동거요인에 대한 지속적인 관찰 연구가 필요한 것으로 사료된다. 생활습관요인 중 음주는 남성의 경우‘평생 안마심’과 ‘음주하다 금주’한 경우가 현재 음주 집단보다 인지기능이 낮았는데, 여성은‘현재음주’집단이 인지기능점수가 가장 낮아 (22.33±5.25) 음주의 영향이 성별에 따라 다르게 나타나는 것으로 보인다. 음주와 인지기능과 관련하여 Park과 Song[8], Shin et al. [23]과Kim과Shim [24]은 음주와 인지기능과 관련이 있는 것으로 보고하고 있으나 Park 등 [10], Topiwala 등[25]은 체계적인 문헌고찰을 통해 음주가 치매와 연관성이 없다고 하였다. 본 결과로도 음주가 인지기능에 미치는 영향이 명확하지 않으므로 이에 대해서는 음주의 정도, 음주기간과 관련하여 조사하고 다른 혼란변수와의 관계를 연구해 볼 필요가 있다고 생각된다. 운동은 남녀 모두에서 운동을 시행하는 군의 인지기능이 높아 신체활동이 인지기능 저하에 미치는 다수의 선행연구[17, 21]와 일치하여 운동이 인지기능 보호요인임을 확인하였다.

반면 사회활동 수, 종교, 고혈압, 당뇨, 고지혈증, 뇌졸중, 질병 수, 흡연유무에 따라서는 전체집단의 결과와 성별 내에의 결과와 차이를 보였는데 본 연구와 같이 성별에 따라 결과를 보고한 선행연구를 찾기 어려워 다른 연구결과와 다각도로 비교하는데 어려움이 있다. 고혈압, 당뇨 등 혈관성 위험요인은 인지기능 저하 진행과 경도인지장애로부터 치매로 이행하는데 있어 위험요인으로 추정되고 있는데[6, 21, 26] 본 연구결과에서는 당뇨유무에 따라서는 인지기능의 차이가 없었고 고혈압, 뇌졸중의 경우에도 전체집단에서는 차이가 없는 것으로 나타나 선행연구[8, 17, 21]와 차이를 보였다. 고혈압정도, 유병기간에 대한 상세한 조사를 통한 추후연구가 필요하다고 생각된다. 고지혈증 유무는 여성에서만 인지기능의 차이를 보였는데 고지혈증이 있는 경우가 없는 경우 보다 더 높아 여성에 있어서는 인지기능에 긍정적인 영향을 미치는 것으로 보인다.

https://doi.org/10.4178/epih.e2017054

Park 등[17]의 연구결과에서도 고지혈증이 음의 상대위험비를 나타내 경도인지장애의 보호요인으로 보고하고 있어 콜레스테롤이 낮거나 체질량지수가 낮은 것은 인지기능장애 위험요인으로 작용한다는 Vidoni 등[27]의 결과와 유사하였다.

뇌졸중유무에 따라서 전체집단과 여성에서는 유의한 차이를 보이지 않았으나 남성에게서만 인지기능의 유의한 차이를 보여(F=5.23, p<.05) 남성에서 위험요인으로 나타났는데 이는 Park 등[17]의 결과에서 뇌졸중이 남성에게 여성보다 더 큰 상대위험도를 보인 것과 유사한 결과였다.

흡연상태에 따른 인지기능은 각 성별 내에서는 차이가 없는 것으로 나타났을 뿐 아니라 전체집단결과를 볼 때 한번도 피우지 않은 집단이 가장 낮은 점수를 보여 흡연이 인지기능에 부정적 영향을 미치지 않는 것으로 나타났다. 이는 흡연여부에 따라 경도인지장애 노인과 정상 노인간에 인지기능의 차이가 없었다고 보고하고 있는 여러 선행연구[10,17,23]과 유사한 결과이다. 그러나 Kim Rakesh등 [7,21]은 흡연을 인지기능의 위험요인으로 보고하고 있어 흡연의 인지기능영향에 대한 좀더 세밀한 연구가 필요하다.

본 연구에서 나타난 바 인지기능의 성별차이가 단순히 성별에 의한 차이인지 인지기능 관련 변인들의 성별 분포차이, 또는 이들 변수와 성별과의 상호작용의 결과인지를 고찰하여 보고자 한다. 먼저 연령을 통제하였을 때 성별 인지기능 차이는 유의하였으나 교육기간을 통제하였을 때는 성별차이가 없는 것으로 나타났다. 이는 교육기간이 동일할 때 인지기능점수가 성별에 따라 차이가 없다는 것으로 성별 인지기능의 차이는 연령보다 교육기간에 따른 것으로 나타났는데 이는 교육이 인지기능에 가장 큰 영향을 미치는 변인이라고 밝힌 Kim[7], Park 등[10]의 결과와 일치한다. Lin et. al [9]은 인지기능 저하와 치매가 남성보다 여성에게 더 영향을 미치는 것으로 보이지만 이는 연령분포의 차이로 나타나는 현상으로 볼 수 있다고 하였다. 그러나 본 연구결과는 교육기간이 낮을수록 인지자극이 부족하여 인지기능장애가 빨리 진행한다는 설명에 해석의 근거를 둘 수 있으며[28], Petersen 등[29]이 남성이 여성보다 상대적으로 높은 교육기간으로 인해 경도인지장애에서 치매로 이행하는 속도가 여성보다 상대적으로 느리다고 한 것과 맥락을 같이 한다.

또한 본 연구에서 사용된 인지기능 관련 변수들 중에서 연령, 교육기간, 사회활동, 동거형태, 음주, 질병 수는 인지기능에 영향을 미침에 있어 성별과 상호작용을 보였으나 연령을 통제한 상태에서는 사회활동, 동거형태의 성별의 상호작용이 보이지 않고 유의한 주 효과만 나타난 것으로 보아 성별 연령의 차이가 사회활동이나 동거형태와 관련되어 인지기능에 영향을 미쳤다는 것으로 짐작할 수 있다. 반면 음주와 질병 수는 연령을 통제하여도 성별과 상호작용이 유의하게 나타났으나 교육을 통제하였을 때에는 성별과의 상호작용이 나타나지 않고 각 변수의 주효과는 유의하게 이들 변수들이 성별과 상호작용이 나타난 것은 성별

교육기간의 차이에서 기인 한 것으로 해석된다.

마지막으로 각 성별 집단 내에서 인지기능에 큰 영향을 미치는 것으로 확인된 교육과 나이를 통제한 상태에서 나머지 변수(연령, 성별, 우울, 교육기간, 경제수준, 동거형태, 과거직업, 사회활동 수, 종교, 당뇨, 고혈압, 뇌졸중, 고지혈증, 보유질환 수, 음주, 흡연, 운동)들이 인지기능에 미치는 영향을 확인한 결과(Table 4) 남녀모두 교육, 나이에 이어 우울이 큰 영향을 미치는 것으로 나타났다. 우울에 이어 인지기능에 유의한 영향을 미친 변수들은 배우자 외의 사람과 동거, 사회활동 수, 경제수준, 운동상태, 종교 유무였는데 이러한 결과는 남, 여 모두 공통적이었고 순서도 비슷하였다. 남녀간에 다르게 나타난 변수는 고지혈증 유무로 여성에서만 유의한 변수로 나타났다. 고지혈증 자체가 인지기능에 보호기능을 하는 것인지, 아니면 연령 외의 교육기간, 경제수준등과의 관련이 있는지에 대해 추후연구를 통해 확인할 필요가 있다.

결론적으로 본 연구결과 인지기능은 성별에 따라 차이를 보였는데 이는 교육기간의 차이에 기인된 것으로 확인되었다. 본 연구에서 사용한 인지기능 영향요인들 중 성별과의 상호작용을 나타낸 변수들은 연령, 교육기간, 사회활동, 동거형태, 질병 수, 음주이었으나 교육을 통제한 상태에서는 모든 변수에서 성별상호작용이 나타나지 않아 이들 변수들이 성별과 상호작용을 보인 것은 교육기간의 차이에 기인한 것으로 보인다. 또한 회귀분석결과 남녀 모두에서 교육, 연령, 우울이 인지기능에 가장 큰 영향을 미치는 중요한 변수였고 이어서 동거형태, 경제수준, 운동, 사회활동, 종교, 뇌졸중유무가 인지기능에 영향을 미쳤는데 이 같은 결과는 남녀 각 집단에서 같았다. 다만 여성에게는 고지혈증이 인지기능영향이 미치는 요인으로 추가되어 이들 요인들은 성별을 고려할 필요가 있다고 생각된다.

추후 연구에서는 인지기능에 영향을 미치는 요인들과 교육과의 상호작용, 또는 병합효과를 확인하는 연구가 이루어질 필요가 있다고 사료된다. 본 연구결과에 의하면 인지기능은 성별에 따른 차이보다 연령과 교육기간에 따른 영향이 큼으로 인지기능을 향상시키기 위한 중재 시 교육기간과 연령을 구분을 하여 프로그램 진행시키는 것이 효과가 높을 것으로 사료된다.

## REFERENCES

1. Korean Statistics Information Service. Estimated future population;2017 [cited 2017 Oct 30]. Available from: http://kosis.kr/statHtml/statHtml.do?orgId=101&tblId=DT_1BPA002&conn_path=I2 (Korean).
2. Han J, Kang H. The relationship between functional physical fitness and mild cognitive impairment in older adults. Korean J Sports Med 2015;33:102-109 (Korean).
3. Kim KW, Kim YJ, Kim, BN, Kim HN, Choi HJ, You JY, et al. The3rd dementia management integration plant. Seongnam: Seoul National University Bundang Hospital; 2015, p. 25-26 (Korean).
4. Prince M, Bryce R, Albanese E, Wimo A, Ribeiro W, Ferri CP. The global prevalence of dementia: a systematic

review and meta analysis. Alzheimers Dement 2013;9:63-75.
5. Ministry of Health and Welfare. 3rd comprehensive plan for dementia management (16'-20'); 2015 [cited 2017 Jul 15]. Available from http://www.mohw.go.kr/react/jb/sjb030301vw.jsp?PAR_MENU_ID=03&MENU_ID=0319&CONT_SEQ=330876&page=1(Korean).
6. Li W, Wang T. Xiao S. Type 2 diabetes mellitus might be a risk factor for mild cognitive impairment progressing to Alzheimer's disease. Neuropsychiatr Dis Treat 2016;12:2489-2495.
7. Kim EJ. Factors influencing cognitive impairment of the elderly residents. J East West Nurs Res 2010;16:122-130 (Korean).
8. Park HK, Song HJ. Predictors of cognitive function decline of elderly: using living conditions and welfare needs of older Korean persons panel data. Korean J Health Serv Manag 2016;10:147-158(Korean).
9. Lin JS, O'Connor E, Rossom RC, Perdue LA, Burda BU, Thompson M, et al. Screening for cognitive impairment in older adults: an evidence update for the U.S. Preventive Services Task Force[cited 2017 May 2]. Available from: https://www.ncbi.nlm.nih.gov/pubmedhealth/PMH0063382/.
10. Park JS, Lee YW, Kim HS. Cognitive impairment risk factors inelders. J Korean Gerontol Nurs 2015;17:121-130 (Korean).
11. Barnes DE, Yaffe K. The projected effect of risk factor reductionon Alzheimer's disease prevalence. Lancet Neurol 2011;10:819-828.
12. Oh E, Lee AY. Mild cognitive impairment. J Korean Neurol Assoc2016;34:167-175 (Korean).
13. Koran ME, Wagener M, Hohman TJ; Alzheimer's Neuroimaging Initiative. Sex differences in the association between AD biomarkers and cognitive decline. Brain Imaging Behav 2017;11:205-213.
14. Kim KW, Kim MH, Kim BJ, Kim JR, Kim TH, Moon SW, et al. Standardization of dementia diagnosis tool. Seongnam: Seoul National University Bundang Hospital; 2009, p. 29-53 (Korean).
15. Yesavage JA, Sheikh JI. Geriatric depression scale (GDS): recent evidence and development of a shorter version. Clin Gerontol1986;5:165-173.
16. Ki BS. A preliminary study for the standardization of geriatric depression scale short form-Korea version. J Korean Neuropsychiatr Assoc 1996;35:298-307 (Korean).
17. Park M, Sung MR, Kim SK, Lee DY. Comparison of demographic characteristics, comorbidity, and health habits of older adults with mild cognitive impairment and older adults with normal cognitive function. J Korean Acad Nurs 2014;44:351-360 (Korean).
18. Park HJ. Factors analysis and prediction models of MCI using the KLoSA [dissertation]. Busan: Pusan National University; 2016(Korean).
19. Gomez-Pinilla F, Hillman C. The influence of exercise on cognitive abilities. Compr Physiol 2013;3:403-428.
20. Boyle PA, Willson RS. Risk factors for Alzheimer's disease. In: Ames D, O'Brien JT, Burns A, editors. Dementia, fifth edition. New York: CRC Press; 2017, p. 447-452.
21. Rakesh G, Szabo ST, Alexopoulos GS, Zannas AS. Strategies for dementia prevention: latest evidence and implications. Ther Adv Chronic Dis 2017;8:121-136.
22. Fratiglioni L, Paillard-Borg S, Winblad B. An active and socially integrated lifestyle in late life might protect against dementia. Lancet Neurol 2004;3:343-353.
23. Shin KR, Kang Y, Jung D, Kim M, Kim J, Kim M, et al. Prevalence and characteristics of mild cognitive impairment in the community-dwelling elderly compared to elderly with normal cognitive function. Korean J Adult Nurs 2011;23:40-49 (Korean).
24. Kim YO, Shim MS. Cognitive functions, instrumental activities of daily living, depression and quality of life in the elderly with mild cognitive impairment. J Korean Public Health Nurs 2015;29:219-230 (Korean).
25. Topiwala A, Allan CL, Valkanova V, Zsoldos E, Filippini N, SextonC, et al. Moderate alcohol consumption as risk factor for adverse brain outcomes and cognitive decline: longitudinal cohorts tudy. BMJ 2017;357:j2353.
26. Schneider BC, Gross AL, Bangen KJ, Skinner JC, Benitez A, GlymourMM, et al. Association of vascular risk factors with cognition in a multiethnic sample. J Gerontol B Psychol Sci Soc Sci 2015;70:532-544.
27. Vidoni ED, Townley RA, Honea RA, Burns JM; Alzheimer's Disease Neuroimaging Initiative. Alzheimer disease biomarkers are associated with body mass index. Neurology 2011;77:1913-1920.
28. Ganguli M, Snitz BE, Lee CW, Vanderbilt J, Saxton JA, Chang CC. Age and education effects and norms on a cognitive test battery from a population-based cohort: the Monongahela-Youghiogheny Healthy Aging Team. Aging Ment Health 2010;14:100-107.
29. Petersen RC, Roberts RO, Knopman DS, Geda YE, Cha RH, Pankratz VS, et al. Prevalence of mild cognitive impairment is higher in men. The Mayo Clinic Study of Aging. Neurology 2010;75:889-897.

Volume: 39, Article ID: e2017054

https://doi.org/10.4178/epih.e2017054

Table 1. Distribution of cognitive-related variables by gender

| Variables | Categories | Total | Men | Women | $X^2$ | p |
|---|---|---|---|---|---|---|
| Gender | | 4878(100.0) | 1110(22.8) | 3768(77.2) | | |
| Age | Below 74 | 1868(38.3) | 475(42.8) | 1393(37.0) | 723.30 | p<0.001 |
| | 75-84 | 2351(48.2) | 519(46.8) | 1832(48.6) | | |
| | Above 85 | 659(13.5) | 116(10.5) | 543(14.40) | | |
| Education period(yr) | 0-3 years | 1803(37.0) | 113(10.2) | 1690(44.9) | 13.58 | p<0.001 |
| | 4-6 years | 1519(31.1) | 321(28.9) | 1198(31.8) | | |
| | 7-12 years | 1365(28.0) | 552(49.7) | 813(21.6) | | |
| | Above 13 years | 188(3.9) | 124(11.2) | 64(1.7) | | |
| Depression | Normal | 3664(75.1) | 830(74.8) | 2834(75.2) | 1.66 | 0.44 |
| | Mild depression | 638(13.1) | 138(12.4) | 500(13.3) | | |
| | Severe depression | 576(11.8) | 142(12.8) | 434(11.5) | | |
| Past occupation | Never | 2819(57.8) | 179(16.1) | 2640(70.1) | 1022.60 | p<0.001 |
| | Yes | 2059(42.2) | 931(83.9) | 1128(29.9) | | |
| No. of social activities | 0 | 1026(21.0) | 335(30.2) | 691(18.3) | 92.69 | p<0.001 |
| | 1 | 2972(60.9) | 640(57.7) | 2332(61.9) | | |
| | More than 2 | 880(18.0) | 135(12.2) | 745(19.8) | | |
| Religion | No | 1665(33.0) | 533(46.2) | 2636(30.0) | 127.98 | p<0.001 |
| | Yes | 3213(67.0) | 577(53.8) | 574(70.0) | | |
| Cohabitant | Alone | 1514(31.0) | 209(18.8) | 1305(34.6) | 593.98 | p<0.001 |
| | Spouse | 1917(39.3) | 781(70.4) | 1136(30.1) | | |
| | Non-spouse | 1447(29.7) | 120(10.8) | 1327(35.2) | | |
| Economic status | National health insurance | 4604(94.4) | 1031(92.9) | 3573(94.8) | 6.28 | 0.01 |
| | Medical Aid | 273(05.6) | 79(07.1) | 194(05.1) | | |

Volume: 39, Article ID: e2017054

https://doi.org/10.4178/epih.e2017054

| | | | | | | |
|---|---|---|---|---|---|---|
| Hypertension | No | 2030(41.6) | 575(51.8) | 1455(38.6) | 61.06 | p<0.001 |
| | Yes | 2845(58.4) | 535(48.2) | 2310(61.3) | | |
| DM | No | 3664(75.1) | 839(75.6) | 2825(75.0) | 0.17 | 1.00 |
| | Yes | 1214(24.9) | 271(24.4) | 943(25.0) | | |
| Hyperlipidemia | No | 4084(83.7) | 1008(90.8) | 3076(81.6) | 52.98 | p<0.001 |
| | Yes | 794(16.3) | 102(9.2) | 692(18.4) | | |
| Stroke | No | 4641(95.1) | 1052(94.8) | 3589(95.2) | 0.42 | 0.52 |
| | Yes | 237(04.9) | 58(05.2) | 179(4.7) | | |
| No. of cormobid diseases | 0 | 833(17.1) | 255(23.0) | 578(15.3) | 51.78 | p<0.001 |
| | 1-2 | 3209(65.8) | 716(64.5) | 2493(66.2) | | |
| | 3 or more | 836(17.1) | 139(12.5) | 697(18.5) | | |
| Alcohol drinking | Never | 3618(74.2) | 239(21.5) | 3379(89.7) | 2105.34 | p<0.001 |
| | Ceased drinking | 552(11.3) | 420(37.8) | 132(3.5) | | |
| | Currently drinking | 708(14.5) | 451(40.6) | 257(6.8) | | |
| Smoking | Never | 3995(81.9) | 329(29.6) | 3666(97.3) | 2659.31 | p<0.001 |
| | Ceased smoking | 705(14.4) | 641(57.7) | 64(1.7) | | |
| | Currently smoking | 178(03.6) | 140(12.6) | 38(1.0) | | |
| Exercise | No | 2264(46.4) | 418(37.7) | 1846(49.0) | 44.28 | p<0.001 |
| | Yes | 2614(53.6) | 692(62.3) | 1922(51.0) | | |

Values are presented as number (%)

ES, Economic status = Health insurance type; DM, Diabetes Mellitus

https://doi.org/10.4178/epih.e2017054

Table 2. Gender comparison of cognitive function score differences by variables

| Variables | Categories | Total | | Men | | Women | |
|---|---|---|---|---|---|---|---|
| | | M(±SD) | t or F(*p*) | M(±SD) | t or F(*p*) | M(±SD) | t or F(*p*) |
| Gender | Men | 25.53(±3.72) | 54.31*** | | | | |
| | Female | 23.76(±4.47) | | | | | |
| Age | Below 74 | 25.17(±3.41) | 303.29*** | 26.32(±3.20) | 25.161*** | 25.56(±2.46) | 202.99*** |
| | 75-84 | 26.64(±4.32) | | 25.04(±3.84) | | 23.25(±4.37) | |
| | Above 85 | 23.16(±4.60) | | 24.25(±4.34) | | 20.86(±5.06) | |
| Education period | 0-3 years | 21.66(±4.49) | 458.35*** | 22.73(±4.15) | 49.99*** | 21.58(±4.5) | 342.63*** |
| | 4-6 years | 24.71(±3.94) | | 24.53(±4.28) | | 24.76(±3.84) | |
| | 7-12 years | 26.47(±2.89) | | 26.40(±2.97) | | 26.52(±2.84) | |
| | Above 13 years | 26.92(±2.66) | | 25.53(±3.72) | | 27.13(±2.82) | |
| Depression | Normal | 24.62(±4.00) | 95.83*** | 26.00(±3.18) | 29.00*** | 24.21(±4.12) | 73.89*** |
| | Mild | 23.23(±4.68) | | 24.43(±4.43) | | 22.89(±4.69) | |
| | Severe | 22.00(±5.67) | | 23.69(±5.16) | | 21.45(±5.72) | |
| Past occupation | Never | 23.40(±4.64) | 208.76*** | 24.53(±4.69) | 15.71*** | 23.33(±4.63) | 83.18*** |
| | Yes | 25.20(±3.73) | | 25.73(±3.47) | | 24.76(±3.88) | |
| No. of social activities | 0 | 23.90(±4.95) | 2.70 | 24.84(±4.23) | 5.78*** | 23.44(±5.20) | 2.85 |
| | 1 | 24.26(±4.24) | | 25.86(±3.33) | | 23.82(±4.36) | |
| | Above 2 | 24.13(±4.07) | | 25.71(±3.92) | | 23.84(±4.04) | |
| Religion | No | 24.29(±4.43) | 10.58*** | 25.53(±4.09) | 0.03 | 24.02(±4.13) | 8.56** |
| | Yes | 23.61(±4.44) | | 25.54(±3.68) | | 23.13(±4.69) | |
| Cohabitant | Alone | 23.99(±4.14) | 206.27*** | 25.35(±3.54) | 17.62*** | 23.78(±4.19) | 130.93*** |
| | Spouse | 25.51(±3.55) | | 25.86(±3.45) | | 25.27(±3.59) | |
| | Non-spouse | 22.55(±4.98) | | 23.74(±5.00) | | 22.44(±4.97) | |

Volume: 39, Article ID: e2017054

https://doi.org/10.4178/epih.e2017054

| | | | | | | | |
|---|---|---|---|---|---|---|---|
| Economic status[1] | National heal insurance | 24.25(±4.27) | 38.17*** | 25.64(±3.58) | 12.98*** | 23.85(±4.37) | 33.23*** |
| | Medical Aid | 22.58(±5.60) | | 24.09(±5.02) | | 21.96(±5.72) | |
| Hypertension | No | 24.33(±4.38) | 5.07* | 25.46(±3.69) | 0.43 | 23.88(±4.54) | 1.79 |
| | Yes | 24.04(±4.36) | | 25.61(±3.76) | | 23.68(±4.14) | |
| DM | No | 24.17(±4.38) | 0.12 | 25.50(±3.79) | 0.33 | 23.78(±4.47) | 0.29 |
| | Yes | 24.13(±4.34) | | 25.65(±3.51) | | 23.69(±4.46) | |
| Hyperlipidemia | No | 23.98(±4.45) | 41.59*** | 25.48(±3.72) | 2.33 | 23.49(±4.56) | 58.98*** |
| | Yes | 25.07(±3.83) | | 26.07(±3.73) | | 24.93(±3.83) | |
| Stroke | No | 24.18(±4.36) | 1.46 | 25.59(±3.70 | 5.23* | 23.76(±4.45) | 0.16 |
| | Yes | 23.83(±4.63) | | 24.45(±3.97) | | 23.63(±4.82) | |
| No. of comorbid diseases | 0 | 24.37(±4.45) | 0.81 | 25.25(±3.73) | 12.97*** | 23.98(±4.68) | 33.23*** |
| | 1-2 | 24.11(±4.36) | | 26.70(±3.61) | | 23.62(±4.45) | |
| | Above 3 | 24.18(±4.33) | | 25.09(±4.35) | | 24.00(±4.30) | |
| Alcohol drinking | Never | 23.84(±4.47) | 45.13*** | 25.15(±4.23) | 6.84** | 23.75(±4.47) | 10.85*** |
| | Ceased drinking | 24.53(±4.36) | | 26.03(±3.30) | | 24.55(±3.72) | |
| | Currently drinking | 25.49(±3.53) | | 25.22(±3.79) | | 22.33(±5.25) | |
| Smoking | Never | 23.90(±4.41) | 40.43*** | 25.37(±3.75) | 0.43 | 23.77(±4.44) | 0.66 |
| | Ceased smoking | 25.42(±3.92) | | 25.61(±3.72) | | 23.56(±5.24) | |
| | Currently smoking | 25.02(±4.21) | | 25.57(±3.68) | | 22.97(±5.35) | |
| Exercise | No | 23.29(±4.78) | 172.78*** | 24.68(±4.41) | 36.94*** | 22.98(±4.80) | 113.62*** |

Volume: 39, Article ID: e2017054

https://doi.org/10.4178/epih.e2017054

| | | | |
|---|---|---|---|
| Yes | 24.91(±3.83) | 26.05(±3.13) | 24.51(±3.98) |

ES, Economic status = Health insurance type; DM, Diabetes Mellitus

*p<0.05, **p<0.01, ***p<0.001

Table 3. Gender interaction in the effect of variables on cognitive function

| Variables | Source of variation | Gender*Variable F | CV: Age Gender*Variable F | CV: Education Gender*Variable F |
|---|---|---|---|---|
| Age | (CV) Age | - | 687.45*** | - |
| | (CV) Education | - | - | 683.20*** |
| | Age | 10.14*** | - | 6.31*** |
| | Gender | 44.68*** | 130.18*** | 2.56 |
| | Gender*Age) | 1.86** | - | 1.11 |
| Education period | (CV) Age | - | 270.07*** | - |
| | (CV) Education | - | - | 1099.20*** |
| | Education | 153.55*** | 104.97*** | - |
| | Gender | 4.03 | 2.98 | 0.09 |
| | Gender*Education | 3.56* | 1.82 | - |
| Depression | (CV) Age | - | 743.92*** | - |
| | (CV) Education | - | - | 1038.37*** |
| | Gender | 60.68*** | 53.46*** | 3.28 |
| | Depression | 10.93*** | 14.30*** | 8.03*** |
| | Gender*Depression | 1.03 | 0.91 | 1.17 |
| Past occupation | (CV) Age | - | 599.32*** | - |
| | (CV) Education | - | - | 1017.58*** |
| | Gender | 32.58*** | 41.15*** | 5.81* |
| | Past occupation | 47.94*** | 17.35*** | 21.06*** |
| | Gender*Past occupation | .39 | 1.16 | 0.17 |
| No. of social activities | (CV) Age | - | 774.23*** | - |
| | (CV) Education | - | - | 1097.86*** |
| | Gender | 2.96 | 6.08* | 1.23 |

| | | | | |
|---|---|---|---|---|
| | No. of social activities | 2.99*** | 8.00*** | 4.82*** |
| | Gender* No. of social activities | 1.94** | 1.17 | 1.38 |
| Religion | (CV) Age | – | 700.72*** | – |
| | (CV) Education | – | | 1063.98*** |
| | Gender | 40.60*** | 40.90*** | 0.15 |
| | Religion | 7.86*** | 9.89*** | 5.84*** |
| | Gender*Religion | 2.11 | 1.64 | 2.12 |
| Cohabitant | (CV) Age | – | 453.65*** | – |
| | (CV) Education | – | – | 896.60*** |
| | Gender | 40.85*** | 44.52*** | 3.69*** |
| | Cohabitant | 66.10*** | 23.90*** | 32.10*** |
| | Gender*Cohabitant | 4.15* | 0.04 | 0.86 |
| ES | (CV) Age | – | 677.74*** | – |
| | (CV) Education | – | | 1108.41*** |
| | Gender | 43.74*** | 26.52*** | 0.30 |
| | ES | 33.86*** | 34.19*** | 39.40*** |
| | Gender*ES | 3.18 | 0.52 | 0.45 |
| Hypertension | (CV) Age | – | 697.34*** | |
| | (CV) Education | – | | 1095.40*** |
| | Gender | 140.78*** | 137.48*** | 0.05 |
| | Hypertension | .038 | 5.33* | 0.51 |
| | Gender*Hypertension | 1.40 | 0.89 | 0.01 |
| Hyperlipidemia | (CV) Age | – | 657.99*** | – |
| | (CV) Education | – | – | 1079.49*** |
| | Gender | 42.48*** | 32.99*** | 1.03 |
| | Hyperlipidemia | 17.75*** | 6.90** | 12.20*** |
| | Gender*Hyperlipidemia | 3.08 | 4.28* | 3.06 |

| | | | | |
|---|---|---|---|---|
| Stroke | (CV) Age | - | 689.25*** | - |
| | (CV) Education | - | - | 1098.46*** |
| | Gender | 15.76*** | 16.28*** | 0.90 |
| | Stroke | 3.69 | 5.87* | 3.81 |
| | Gender* Stroke | 2.27 | 1.26 | 0.84 |
| No. of comorbid disease | (CV) Age | - | 696.34*** | - |
| | (CV) Education | - | - | 1097.85*** |
| | Gender | 105.45*** | 93.99*** | 0.78 |
| | No. of comorbid diseases | 0.17 | 2.07 | 0.56 |
| | Gender*No. of comorbid diseases | 2.74* | 3.57* | 2.12 |
| Alcohol drinking | (CV) Age | - | 667.61*** | - |
| | (CV) Education | - | - | 1093.93*** |
| | Gender | 87.52*** | 95.52*** | 1.01 |
| | Alcohol drinking | 16.39*** | 6.52*** | 14.83*** |
| | Gender*Alcohol drinking | 4.62** | 4.08* | 3.01 |
| Smoking | (CV) Age | - | 689.75*** | - |
| | (CV) Education | - | - | 1100.74*** |
| | Gender | 38.97*** | 29.03*** | 1.04 |
| | Smoking | 0.27 | 1.60 | 0.67 |
| | Gender*Smoking | 0.89 | 0.01 | 1.52 |
| Exercise | (CV)Age | - | 616.97*** | - |
| | (CV) Education | - | - | 1003.71*** |
| | Gender | 119.45*** | 107.50*** | 0.27 |
| | Exercise | 94.69*** | 61.56*** | 46.25*** |
| | Gender*Exercise | 0.31 | 0.11 | 0.01 |

CV, co-variable ES, Economic status = Health insurance type; DM, Diabetes Mellitus

*p<0.05, **p<0.01, ***p<0.001

Table 4.Factors influencing cognitive function according to gender

| Gender | Variables | Model 1 | | | | | Model 2 | | | | |
|---|---|---|---|---|---|---|---|---|---|---|---|
| | | B | SE | β | t | p | B | SE | β | t | p |
| Men | (Constant) | 28.93 | 1.26 | | 23.04 | p<.001 | 28.39 | 1.40 | | 20.28 | p<.001 |
| | Education period | 1.38 | 0.13 | 0.30 | 10.65 | p<.001 | 1.20 | 0.13 | 0.26 | 9.44 | p<.001 |
| | Age | -.092 | 0.02 | -0.17 | -6.10 | p<.001 | -0.10 | 0.02 | -0.19 | -6.51 | p<.001 |
| | Depression | | | | | | -0.17 | 0.03 | -0.16 | -5.43 | p<.001 |
| | Cohabitant: Not spouse | | | | | | -1.32 | 0.33 | -0.11 | -3.99 | p<.001 |
| | No. of social activities | | | | | | 0.56 | 0.16 | 0.10 | 3.51 | p<.001 |
| | ES | | | | | | -1.23 | 0.41 | -0.09 | -3.03 | .002 |
| | Exercise | | | | | | 0.60 | 0.21 | 0.08 | 2.80 | .005 |
| | Stroke | | | | | | -1.05 | 0.48 | -0.06 | -2.22 | .027 |
| | Religion | | | | | | 0.42 | 0.21 | 0.06 | 2.03 | .043 |
| | | F(p)= 89.35*** | | | | | F(p)= 18.83*** | | | | |
| | | $R^2$=0.14 Adjusted $R^2$=0.14△$R^2$=0.14 | | | | | $R^2$=0.23 Adjusted $R^2$=0.21 △$R^2$=0.09 | | | | |
| Women | (Constant) | 31.39 | 0.79 | | 39.58 | p<.001 | 31.12 | .90 | | 34.72 | p<.001 |
| | Education period | 1.92 | 0.08 | 0.36 | 23.28 | p<.001 | 1.72 | .08 | .321 | 20.96 | p<.001 |
| | Age | -0.14 | 0.01 | -0.24 | -15.43 | p<.001 | -.14 | .01 | -.229 | -13.38 | p<.001 |
| | Depression | | | | | | -.21 | .02 | -.157 | -10.93 | p<.001 |
| | Cohabitant: not spouse | | | | | | -.74 | .17 | -.079 | -4.39 | p<.001 |
| | ES | | | | | | -1.30 | .28 | -.065 | -4.65 | p<.001 |
| | Hyperlipidemia | | | | | | .63 | .19 | .054 | 3.29 | .001 |
| | No. of social activities | | | | | | .31 | .10 | .045 | 3.08 | .002 |
| | Exercise | | | | | | .33 | .13 | .037 | 2.55 | .011 |
| | Religion | | | | | | -.33 | .14 | -.033 | -2.37 | .018 |
| | Stroke | | | | | | -.63 | .30 | -.030 | -2.09 | .037 |
| | | F(p)=635.99*** | | | | | F(p)=99.00*** | | | | |

| | | |
|---|---|---|
| | $R^2$=0.25 Adjusted $R^2$=0.25 △$R^2$=0.25 | $R^2$=0.31 Adjusted $R^2$=0.31△$R^2$=0.06 |

ES, Economic status

***p<0.001
